# The cardio-oncology multidisciplinary team: beyond the basics

**DOI:** 10.1186/s40959-025-00369-8

**Published:** 2025-07-26

**Authors:** Joshua D. Bennetts, Trent D. Williams, Craig J. Beavers, Heather N. Moore, Cameron Robson, Thomas Warner, Susan Dent, Aaron L. Sverdlov, Doan T.M. Ngo

**Affiliations:** 1 School of Biomedical Sciences and Pharmacy, University of Newcastle, Callaghan, NSW 2308 Australia; 2 Hunter Medical Research Institute, New Lambton Heights, Newcastle, NSW 2305 Australia; 3 Newcastle Centre of Excellence in Cardio-Oncology, Hunter Medical Research Institute, Calvary Mater Newcastle, The University of Newcastle, Hunter New England Health, Newcastle, NSW Australia; 4 Hunter New England Local Health District, New Lambton Heights, NSW 2305 Australia; 5 School of Nursing and Midwifery, University of Newcastle, Callaghan, NSW 2308 Australia; 6 Department of Pharmacy Practice and Science, University of Kentucky College of Pharmacy, Lexington, KY United States of America; 7 Duke Cancer Institute, Durham, NC United States of America; 8 Department of Medicine, Wilmot Cancer Institute, University of Rochester, Rochester, NY USA; 9 School of Medicine and Public Health, University of Newcastle, Callaghan, NSW 2308 Australia; 10 Calvary Mater Newcastle, Waratah, NSW 2298 Australia

**Keywords:** Cardio-Oncology, Pharmacist, Nurse, Multidisciplinary, Medication management, Education, Telemedicine, Rural

## Abstract

A cardio-oncology multidisciplinary team is essential for the successful delivery of patient-centred care. The roles of oncologists, haematologists, and cardiologists have been clearly articulated in literature pertaining to the creation of cardio-oncology clinics. However, the involvement of other key team members, such as pharmacists, nurses and nurse practitioners, social workers, psychologists and other allied health professionals has been less well-defined. In this review we aim to define the role of pharmacists and nurses as part of a multidisciplinary cardio-oncology team. We also discuss models of care and opportunities to expand the delivery of cardio-oncology services to further enhance outcomes for individuals with cancer, and highlight the challenges experienced by those living in regional, rural, and remote communities.

## Background

Cardiovascular disease (CVD) and cancer are the two leading causes of morbidity and mortality in the Western world [1]. Currently, there are over 30 million cancer survivors worldwide [2]. The number of cancer survivors is projected to increase by 30%, with nearly 2 million Australians estimated to be living post cancer by 2040; [3] a result of improved cancer screening, treatments, and the aging population [4]. 

CVD is a leading cause of long-term morbidity and mortality among cancer survivors [5–7] and is an all-too-common consequence of cancer therapy-related cardiovascular toxicity (CTR-CVT); [8] a complication that can be amplified through the presence of pre-existing cardiovascular risk factors including hypertension (HTN), diabetes mellitus (DM), dyslipidaemia, obesity, and advanced age [9]. A number of professional societies including the European Society of Cardiology (ESC) [10], the International Cardio-Oncology Society (IC-OS) [11], the European Society of Medical Oncology (ESMO) [12], and the American Society of Clinical Oncology (ASCO) [13], have published guidelines or position statements that define the importance of baseline cardiovascular (CV) surveillance and care during cancer treatment and throughout patient survivorship. Whilst tremendous international effort has culminated in the formation of the first guideline published by ASCO in 2017 for the management of complex and a diverse range of CTR-CVT [13], there is a clear lack of evidence-based, CV-focussed care pathways for cancer patients from the time of diagnosis to survivorship [14]. 

The need for a multidisciplinary approach to patient care is becoming increasingly apparent as complications secondary to cancer therapy emerge; particularly during the treatment phase of a patient’s cancer journey [15]. The clinical needs of people with cancer are complex, especially considering the intricate interplay between cancer treatment, comorbidities and ageing [16]. As more patients survive a cancer diagnosis, the requirement of new innovative models of care to address these needs remain vital [17, 18]. The cardio-oncology multidisciplinary team (CO-MDT) continues to establish itself as a cornerstone for appropriate prevention, mitigation and management of CV toxicities associated with cancer treatment, whilst providing ongoing support for the treatment of cancer [19, 20]. 

The roles of pharmacy, nursing and allied health are ingrained in clinical practice in all cardiology specialties including heart failure (HF), atrial fibrillation (AF), and cardiac rehabilitation. These personalized models of care have been shown to improve patient outcomes and access to care, are easily adopted, and are economically viable [21–27]. However the role of pharmacy, nursing, and allied health within the CO-MDT has been less clearly defined. It is therefore critically important to create cardio-oncology (CO) care pathways to mitigate CVD development for patients living with, through and beyond cancer [28–30]. In this review, we discuss the proposed roles and responsibilities of pharmacists, nurses, and nurse practitioners, along with other key supportive care providers, as part of the broader CO-MDT.

### The cardio-oncology pharmacist

The role of a pharmacist has been clearly articulated in many clinical settings, which often involves the provision of education and tailored support to patients, medication management solutions, detecting drug-drug and drug-disease interactions, and supporting the safe and effective prescribing of medications [31–36]. Although the need for pharmacists within a CO-MDT is well established in literature describing the development of successful CO clinics [19, 20, 37, 38], their particular role within CO is yet to be completely extrapolated.

The clinical utility of pharmacists within the space of CO clinics — both physical and virtual — is diverse in their support of cardiologists and oncologists in the management of CTR-CVT. A pharmacist’s role varies depending on the size of the institution and the apparent need for their entire scope of practice [20]. Cardiology and oncology pharmacists can assist the broader CO-MDT (see Table 1) by participating in shared clinical decision-making in response to changes in a patient’s CV function, along with formulating and updating cancer treatment-specific pathways based on current available evidence [39]. Appropriate pharmacist-led education on CTR-CVT is also essential for the optimal care of people with cancer [37]. 

The multidisciplinary Team IntervenTion in cArdio-oNcology (TITAN) trial (NCT01621659) established a design for a multidisciplinary CO service [40]. Patients were recruited from a single site at the Cross Cancer Institute, Edmonton, Alberta, Canada [41]. The study aims to determine the effects of a multidisciplinary team approach on the early identification and intervention of CV risk factors to prevent left ventricle (LV) remodeling in people with cancer receiving adjuvant therapy [40, 41]. Within this unit, clinical pharmacists monitor cancer patients’ prescribed medication and provide ongoing monitoring and support where indicated to support early management of CV risk factors during cancer treatment and reduce LV remodelling [40]. The TITAN trial remains active and hence results are unavailable at the time of writing, particularly surrounding the pharmacist assessment and counselling intervention, and treatment of CV risk factors.

The virtual-hybrid CO service proposed by Brown and colleagues highlights the full scope of practice clinical pharmacists deliver within their model of care [42]. This includes discussing and reviewing essential pharmacotherapies to manage comorbidities such as HTN, dyslipidaemia, and CTR-CVT such as HF, as well as commenting on potential drug-drug interactions between anticancer therapies and a patient’s concurrent medication regimen [42]. Pharmacists can also assist with medication titration and prescription, as well as providing medication education and smoking cessation advice for patients [42]. 

**Table 1:** Roles and responsibilities of specialised pharmacists in cardiology and oncology. Nuances exist between the roles of cardiology and oncology/haematology pharmacists. However, the core responsibilities are very similar. pharmacists within these disciplines are at the forefront of medication education, reconciliation, and optimisation for patients

Roles and Responsibilities of Specialised Pharmacists	
*Cardiology Pharmacist* [24, 43]	*Oncology/Haematology Pharmacist* [44, 45]
• Patient education • Drug information • Medication reconciliation and review • Medication optimization and titration • Monitoring and management of adverse effects and drug interactions • Collaborative clinical decision-making, including drug selection and dosing for cardiovascular disease • Pharmacovigilance and medication safety • Implementation and review of local hospital protocols, guidelines, and policies • Dose adjustments based on pharmacokinetics and pharmacodynamics	• Pharmacotherapy management of patients with cancer and coordination of anticancer administration • Patient and staff education • Medication therapy management • Sterile compounding of anticancer treatment • Adverse drug reaction prevention and monitoring • Shared decision-making • Dose adjustments for organ dysfunction, weight, and age • Therapeutic drug monitoring • Laboratory monitoring • Formulation/route of administration optimisation • Medication reconciliation • Assuring medication regimen concordance

**Fig. 1:**
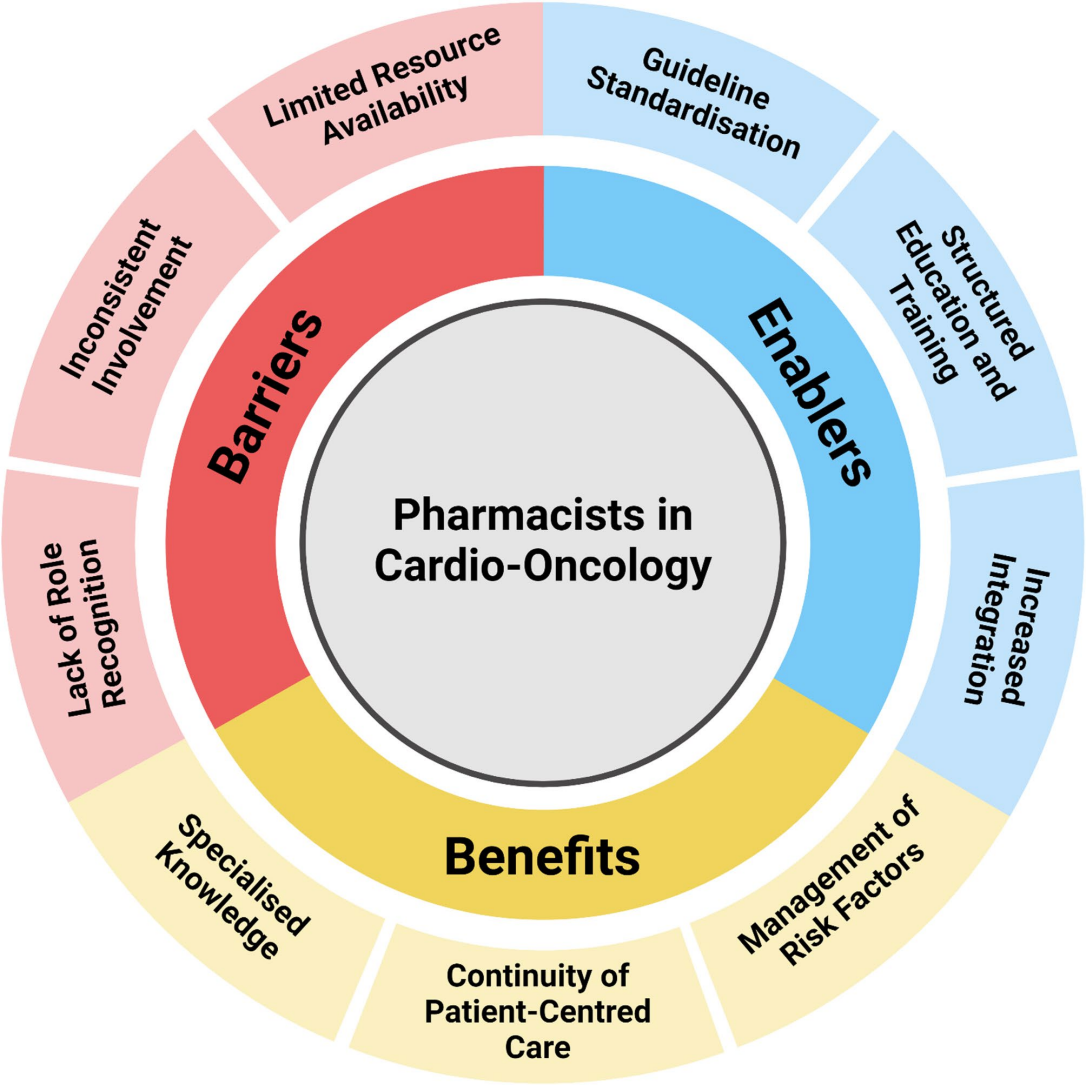
Barriers, enablers, and benefits of inclusion of pharmacists into the cardio-oncology multidisciplinary team. Whilst the inclusion of pharmacists into the cardio-oncology multidisciplinary team is widely accepted, several barriers have been identified that hinder their involvement, including lack of role recognition and resources available to cardio-oncology pharmacists. Postulated solutions to overcome these barriers include integration of pharmacists into cardio-oncology clinics to increase pharmacist exposure, along with the development of structured education or training schemes. This must also be reflected in cardio-oncology guidelines pertaining to the development of multidisciplinary teams. Adapted from Merali A et al. “Exploration of current pharmacy practice in cardio-oncology: Experiences & perspectives”. J Oncol Pharm Pract. 2023;29(8):1844–52

Recently, Merali and colleagues [46] described the perspectives of Canadian pharmacists who provide care for oncological patients at risk of CTR-CVT (Fig. 1). Participants highlighted the benefit of pharmacist involvement in patient care through the utilisation of specialised drug knowledge to enhance patient-centred care. A key challenge identified by these pharmacists was a lack of role recognition, demonstrating the need to define the position of pharmacists more clearly within a CO-MDT.

**Fig. 2:**
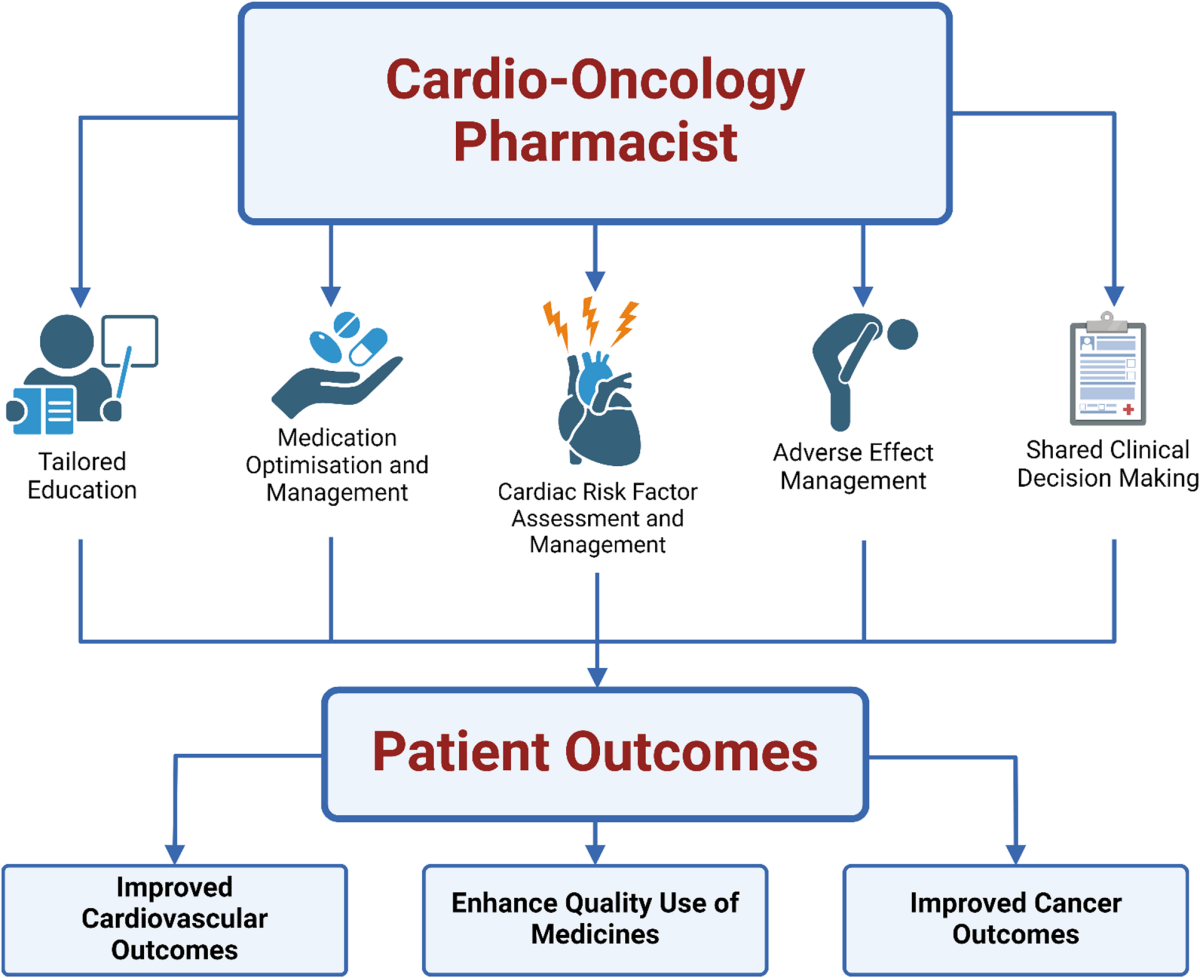
Role of the pharmacist as part of a cardio-oncology multidisciplinary team. Pharmacists are well-positioned to provide a range of medication-centric services to enhance both cancer and cardiovascular outcomes. Such services target optimising medication use and management and providing patient-centred education to improve the quality use of medicines

Patel and colleagues [47] propose a multidisciplinary team-based model for the management of HTN secondary to angiogenesis inhibitors such as sunitinib and bevacizumab. In this model, pharmacists act as ‘liaisons’ between oncology and cardiology teams, as well as ensuring appropriate access to treatment — particularly for oral angiogenesis inhibitors. After the creation of an initial care plan by the oncology team, patients are referred to a pharmacist for a comprehensive baseline CV risk assessment. Subsequent blood pressure goals following this CV risk assessment are incorporated into the patient’s treatment plan. Pharmacists then assist with the optimization of patient-specific risk factors — including pre-existing HTN — prior to commencing anticancer therapy through pharmacological and non-pharmacological interventions. Within this model [47], pharmacists provide patients with drug- and disease-specific education and self-monitoring tools. Follow-up consultations are arranged for pharmacists to assist with the ongoing monitoring of pathologies and blood pressure goals, along with continual medication reconciliation. This provides a referral pathway for patients to cardiology if blood pressure is inadequately managed [47]. Greater clarity surrounding the role of specialised CO pharmacists within a CO-MDT will empower pharmacists to provide tailored support and education to patients with cancer, along with contributing detailed knowledge to the broader MDT, thereby enhancing the quality of care provided during treatment and throughout survivorship (see Fig. 2).

#### Pharmacist medication management in cardio-oncology

Complex medication regimens are associated with poorer medication adherence and a greater likelihood of hospitalization relating to adverse medication-related events [48, 49]. Cancer therapy initiation may then further increase the complexity of a patient’s existing drug regimen, requiring additional medication management support to minimize medication-related harm. Proposed roles of pharmacists within a CO-MDT largely focus on medication reconciliation, as illustrated in Table 2. Current gaps in care identified by cancer patients themselves include gaps in communication amongst stakeholders [50] and understanding the potential adverse impacts of anti-cancer drugs regimes [51]. 

Despite the absence of high-quality evidence, studies demonstrate the clinical utility of pharmacist-led medication reviews (PMRs) in resolving actual and potential medication-related issues, reducing medication-related harm through education, improving medication regimen concordance and enhancing communication between healthcare providers [52, 53]. There are numerous iterations of PMR programs internationally in countries such as Australia and New Zealand, the United Kingdom, the United States of America [54–57]. Whilst the medication review structure may differ, the common core aim across established comprehensive medication review services is to promote prescriber-pharmacist collaboration in the creation of a medication management plan for the patient [53]. The pharmacist provides a detailed report to the patient’s prescriber regarding any real or potential adverse drug events, such as drug-disease interactions or drug-drug interactions (DDIs), absent or inappropriate pharmacotherapies, and medication management difficulties experienced by the patient.

The perspectives of transition-of-care patients and their experiences with pharmacist-led post-discharge medication management and reviews has been previously explored [58]. Perceived benefits included improved health literacy and education provision that lead to a reduction in anxiety associated with medication changes, along with enhancing communication between members of the patient’s healthcare team, such as primary care physicians [58]. Whilst the study population were hospitalized due to a primary diagnosis of CVD rather than cancer, the results can be extrapolated to those with cancer who frequently transition between hospital and home. PMRs could therefore present a useful initiative as part of a cancer patient’s treatment plan and would be a vital component of a specialized CO pharmacist’s role.

#### Significance of drug interactions for the cardio-oncology patient

Interactions are highly prevalent in the context of anticancer treatments, with one study demonstrating that nearly 80% of patients being treated for cancer have at least one potential DDI [59]. This presents a considerable issue in CO. In a recent statement from the American Heart Association (AHA), pharmacists are identified as an essential component of the CO-MDT in addressing the pharmacodynamic and pharmacokinetic interactions that exist between pharmacological therapies for cancer and CVD [60]. 

Pharmacokinetic interactions involving cytochrome P450 (CYP) enzymes and P-glycoprotein (P-gp) efflux pumps are touted as the most common interactions within oncology [61]. Many anticancer agents themselves are inhibitors or inducers of metabolic enzymes that impact the concentrations of not only anticancer therapies, but other pharmacological agents concomitantly administered for the management of comorbidities [62]. Several anticancer agents and CV drugs interact via CYP metabolic pathways, either through direct hepatic drug metabolism or its influence over CYP enzyme activity [63]. A common and important clinical scenario relevant to CO is the prescribing of anticoagulants in cancer patients. For example, the concurrent use of apixaban with idelalisib — a potent inhibitor of CYP3A4 — may lead to a drastic increase in apixaban serum concentration and subsequent increase in bleeding risk. Hence combination should be avoided and an alternative treatment investigated [60]. 

The importance of monitoring for pharmacodynamic DDIs can be illustrated by potential interactions between tyrosine kinase inhibitors (TKIs), such as alectinib — indicated for anaplastic lymphoma kinase (ALK)-positive non-small cell lung cancer — or dasatinib — indicated for chronic myeloid leukemia — and CV medications. Coadministration of lorlatinib with other bradycardic agents such as beta-blockers increases the risk of severe symptomatic bradycardia, hence combination should be avoided [64]. Similarly, combining dasatinib with antiarrhythmic drugs such as sotalol, flecainide, or amiodarone may prolong the QTc interval and increase the risk of torsades de pointes and sudden cardiac death [65]. Identification of DDIs is therefore critical in maintaining appropriate cancer treatment efficacy, whilst minimising the risk of toxicity of both cancer treatment and CV pharmacotherapies [48, 49]. 

#### Pharmacist provision of telemedicine: an area of expansion??

The healthcare’s response to limited physical interaction with patients amongst the height of the COVID-19 pandemic has consequentially paved the way for new models of care. The use of telehealth as a solution demonstrates the feasibility of remote care provision for patients with CVD and cancer [66–70]. However, the concept of telepharmacy predates the rapid growth of telehealth due to the recent pandemic. In 2014, Sankaranarayanan and colleagues [71] performed a retrospective cross-sectional study evaluating a telepharmacy service model by comparing documented pharmacist interventions across eight hospital sites. The study compared the interventions and outcomes between sites with or without an onsite rural hospital pharmacist. Results demonstrated that the introduction of telepharmacy services significantly increased the spectrum of proposed interventions, patient-centered interventions, and health system-centered interventions from baseline. Interventions with both an on-site and off-site (remote) pharmacist were significantly more patient-centric, actionable, and corresponded with transcribing and prescribing of medicines [71]. 

The more recent demand for telemedicine has encouraged the expansion of pharmacy services delivered remotely. Crilly and Kayyali [72] reviewed randomised controlled trials involving telehealth and digital technology-assisted community pharmacist services to better understand how these services are being utilized. Most studies assessed an improvement in public health outcomes such as medication adherence, medication counselling, HTN management, vaccination uptake, and smoking cessation. The most common telehealth service provided by community pharmacists was telephone calls or automated telephonic prompts, however the authors acknowledged the limited use of novel technologies, such as mobile phone applications, and the limited public health topics addressed through telehealth interventions [72]. Provision of telehealth services may enable greater delivery of medication-related services to oncology patients for the management of their CV complications.

### Nursing in cardio-oncology: contribution to the multidisciplinary team

The need for ongoing CV care in cancer patients is articulated in consensus statements and within the literature [9, 12, 73]. However, given the challenges of local availability of staff, differing geography, funding arrangements and different health systems, a “one size fits all approach” remains a challenge.

The role of nursing as a part of a team-based approach in the early identification and management of diseases in many specialties is widely acknowledged [74–76]. Nursing has established efficacy in models of care ingrained in CV patient management including cardiac rehabilitation, chest pain clinics, HF, AF, and HTN [77–81].  A nurse specialist with highly developed clinical skills, commitment to patient education, and management across the full spectrum of the disease process underpins this model of care.

In cardiology settings the improvement in access to care, guideline adherence, treatment outcomes, improved medication adherence, and improved health outcomes have been consistently demonstrated. Furthermore, this multidisciplinary approach is now embedded in international guidelines and consensus statements [82–84]. Importantly, this can be in a variety of settings including hospital settings, outpatient clinics, home based care and now the emergence of telehealth options has increased post discharge follow up support. Similarly, nurse practitioner (NP) models of care in cardiology have demonstrated the potential for similar benefits including reductions in 30-day hospital readmission rates [85, 86] and high patient satisfaction; [87] although most studies assessing NP involvement in cardiology have been of poor quality [88]. 

NPs have played a vital role in oncology MDTs for several decades, with the first oncology NP competencies established in 2007 by the Oncology Nursing Society [89]. These competencies were updated in 2019 to reflect the expanding clinical, education, administration, and policy advancement roles of NPs in cancer care [90]. Oncology NPs are often viewed as care coordinators and patient advocates for people receiving cancer therapy or palliative care [91]. The inclusion of oncology NPs in emergency cancer care services has demonstrated reductions in emergency department presentations and hospital admissions [92–94]. Oncology NPs also provide ongoing cancer surveillance, symptom monitoring and managing and health promotion education as part of post-treatment survivorship programs [95]. 

However, the role of NPs within the CO-MDT has not yet fully been recapitulated, though the acknowledgement of their importance has been described [19, 37, 96, 97]. The instigation of primary prevention strategies can be potentially a challenging time for health professionals in ensuring adherence and patient acceptability, a coordinated systematic multidisciplinary approach can enhance patient care and improve patient outcomes [85, 86, 98]. 

One of the cornerstones of these models is the team-based approach where care co-ordination, patient education, and multidisciplinary collaboration are all features of a strong multidisciplinary care program. The emergence of CO can allow the same principles involved in the assessment, clinical management and patient empowerment of this group of patients to be translated into this subspecialty to enhance patient care (see Table 2). As cancer treatments continue to improve prognosis of cancer patients and enhance outcomes, long term vigilance and support will remain of the upmost importance to this population [99].

Nursing models of care remain an evidenced based option and have a focus on: [100]

*Clinical Assessment* — Blood pressure, pulse, electrocardiogram, non-invasive evaluation, identification of underlying risk factors, care coordination, titration of medications. This includes before and during treatment, and in a survivorship capacity.

*Lifestyle Advice* — Nutritional advice, exercise advice, smoking cessation, weight loss education and the establishment of referral pathways to allied health providers.

*Education* — Providing education on disease process and management, sign and symptom recognition, potential complications, and medication use and self-management support.

Whilst this model has been demonstrated to be effective, there is evidence that there are positive economic benefits to nursing-led models of care. Nurse-led models of care have been shown to be associated with fewer hospital days per year and reflected cost savings [79]. Importantly when combined with the multidisciplinary team, including pharmacists, the results have demonstrated important positive outcomes for chronic disease management in a variety of settings [101, 102]. The early experience of the specialised CO nursing services is extremely positive [103, 104]. Translating this to be accepted internationally, utilizing current models of care remains of upmost importance in delivery care for the cancer patient.

**Table 2:** Studies investigating the roles of pharmacists and nurses as part of a cardio-oncology multidisciplinary team. The importance of both pharmacists and nurses as part of a multidisciplinary team has been well-established. However, literature pertaining to their specific role within a cardio-oncology multidisciplinary team is scarce. However, the limited evidence demonstrates the clinical utility of pharmacists and nurses within a cardio-oncology multidisciplinary team to enhance quality of care and maximise positive patient outcomes. CV, cardiovascular; CVD, cardiovascular disease; CO, cardio-oncology; CO-MDT, cardio-oncology multidisciplinary team; acei/arb, angiotensin converting enzyme inhibitor/angiotensin receptor blocker

Author	Study Design	Cardio-Oncology Clinical Setting or Model of Care	Outcomes/Findings
**Pharmacists within a Cardio-Oncology Multidisciplinary Team**			
**Yaseen** and **Farhan**, [105] 2022	Prospective observational study of 333 cancer patients referred to the Iraqi Cardio-Oncology Program-Pharmacist (iCOP-pharm) program.	Cardiologist and cardiology pharmacist model of care. • Medication reconciliation performed by a pharmacist, providing CV drug interventions (drug titration, switching, cessation, or initiation) • Echocardiography provided by cardiologist • Discussion between pharmacist and cardiologist regarding medical history and drug interventions • Joint decision-making between cardiologist and pharmacist	• 200 cancer patients identified as having established CVD and/or concurrent CV risk factors • Cardiology pharmacist responsible for 75% of total CV drug interventions • CV drug initiation was the most common intervention • Limited by small sample size
**Merali**, et al., [47] 2022	Qualitative telephone interview study of experiences and perspectives of pharmacists on current pharmacy practice in CO.	Survey conducted across multiple regions of Canada, including Ontario, British Columbia, and Alberta. • One pharmacist in community (outpatient/inpatient) • Four pharmacists working in an ambulatory hospital setting • Two pharmacists residing in ambulatory settings (including regional) • One pharmacist working in an ambulatory and hospital inpatient settings	• Pharmacist involvement enhanced patient-centred care and provision of specialised medication-related knowledge • Barriers to optimization of care included lack of role recognition and resources for pharmacists working in CO • Poor communication across healthcare teams impeded continuity of care for cancer patients • Suggested solutions for barriers were the establishment of survivorship programs for long-term follow-up and to increase integration of pharmacists into CO-MDTs • Guidelines or standards of care recommended to standardise delivery of clinical services
**Einsfeld**, et al., [106] 2023	Case study of 15-day CO rotation program for onco-haematology pharmacy residents at a university hospital in Porto Alegre, Brazil.	Two-week program objectives to develop clinical skills in CO: • Understand mechanisms of CV toxicity from cancer treatment • Experience CV assessment facilitated by a cardiologist • Focus on pharmacological management of CVD, particularly heart failure and myocardial damage prevention • Participate in care of patients with CVD, including transitions of care activities	Post-rotation evaluation: • Broad cardiology experience delivering clinical services such as medication reconciliation and outpatient clinics (including CO) • Recommended to expand resident participation in CO clinics Evaluation 2-years after residency: • Expanded knowledge of cardiology • Stimulated interdisciplinarity between oncology and cardiology
**Nurses within a Cardio-Oncology Multidisciplinary Team**			
**Pareek** et al., [107] 2018	Prospective cohort study of 535 cancer patients referred to CO clinic at the Royal Brompton Hospital, London, United Kingdom.	Clinic comprised of consultant cardiologists, senior clinical fellow in CO, and senior clinical nurse specialist. • Supported by specialist cardiac imaging with advanced echocardiography and CV magnetic resonance • Initial one-stop day case service model – expedite CV status assessment and provide rapid opinion to referrers • Baseline clinical assessment: cardiac biomarkers, electrocardiogram, transthoracic echocardiography • Risk of cardiotoxicity classification based on assessment • Lifestyle advice and consultation with clinical nurse specialist	• 238 patients (44.4%) deemed high risk for cardiotoxicity and referred for baseline assessment - Optimisation of cardioprotective medications including beta-blockers and ACEi/ARB - 86% of patients deemed fit for cancer treatment • Clinical activity increased from 105 patients/year to 179 patients/year • Increase in average number of new day case assessment patients seen in weekly clinic (2.3 vs. 3.6 patients/day) • High satisfaction for overall day case experience
**Zhang**, et al., [108] 2023	Qualitative study surveying 739 oncology nurses working in tertiary hospitals in the Shanxi Provence, China.	• Two-part questionnaire: 1) General information regarding hospital settings and nursing practice - Demographic data - Exposure to CO training within their department 2) Evaluation of nurses’ CO knowledge - Multiple choice questions divided into three domains (1) disease observation, (2) health guidance, and (3) implementation of treatment • Questions were credited with 1 point each, with a total of 10 points possible	• Low awareness rate of CO knowledge amongst surveyed nurses - 40.4% of responders could recognise potentially cardiotoxic antineoplastics, with 12.5% able to identify when cardiotoxicity would occur - Low awareness of health guidelines pertaining to oncology patients with heart disease (7.1%) - 44.7% of responders were aware of precautions for cardiotoxicity that nurses can implement • Identified greater need for precision training for nurses in CO to improve quality of care
**Pharmacists and Nurses within a Cardio-Oncology Multidisciplinary Team**			
**Liu**, [109] 2023	Feasibility and acceptability study of a novel CO service line model of care for large, multi-hospital health systems in collaboration with academic cancer centres.	• CO team comprised of cardio-oncologists, vascular oncologists, specialists in advanced heart failure and infiltrative heart disease, CO pharmacists, CO administrative coordinators, and nurse practitioners • No specific description of the role of pharmacist and nurse in CO-MDT	• Feasible and effective model of care for enhancing CO care quality and accessibility • Service line can be used in conjunction with academic CO programs to enhance the efficacy of CO healthcare provision

### The potential importance of specialized models of care for Cardio-Oncology patients in rural areas

Rural patients experience CVD rates at 20% higher compared to metropolitan patients and have worse clinical outcomes [110]. This health outcome disparity is additionally reflected in rural cancer outcomes, with mortality rates 7% higher compared to metropolitan cancer patients; a concern that remains largely unchanged [111]. In addition, given the challenges of access to care and specialty care for rural patients, further clinical pathways involving the multidisciplinary team are needed to guide improvement in risk stratification, management and treatment pathways for all rural cardiology patients [112, 113]. Nursing-led clinics have been shown to enhance access to healthcare in diverse settings [114–116]. Pharmacist involvement in the healthcare provision for rural communities have also been demonstrated to lead to improved patient outcomes across a myriad of disciplines within medicine [117–119]. Improvement in the management of CV care, by strengthening clinical pathways, utilising multidisciplinary teams of cancer patients may have the potential for improved care of rural cancer patients.

### The next steps: developing Cardio-Oncology pharmacists and nurses

There is limited literature pertaining to education and training for pharmacists and nurses in the field of CO. Whilst there is a general acknowledgement of the need for formalized training to enhance the delivery of CO care to people with cancer [120, 121], there has yet to be the proper translation of these education initiatives into practice. Greater intentional training of pharmacists and nurses within a CO-MDT will help improve communication between the two traditionally siloed disciplines of cardiology and oncology, improving cancer outcomes and survivorship programs, and growing future leaders within CO.

Most training models focus on bridging the knowledge gaps between cardiology, oncology and haematology for physicians, which includes clinical rotations and fellowships [120–122]. However, there is a growing need for structured programs that bridge this gap for other healthcare providers to create a true CO-MDT. Brown and colleagues [123] describe the core components required for training and career development in CO applicable to pharmacists, nurses, and physicians. Included in their proposition are four key components: (i) adequate infrastructure to support learning, (ii) fellowship opportunities in CO, (iii) output demonstrating the results and impact from different training models, and (iv) specialty development, such as through continuing education targeting the broader CO-MDT.

An ideal model of training and education for pharmacists and nurses would include a combination of formal education through structured CO coursework, providing fundamental knowledge, and fellowships to work as part of a CO-MDT within developed CO clinics [123]. IC-OS provides health professionals access to a myriad of CO-related educational resources and guidelines, which includes a cardio-oncology nursing education program tailored to the specific needs of nurses providing CO services [124]. The nursing program involves a series of modules covering cardiovascular care, cancer therapeutics, cardiotoxicities and their management, and cancer survivorship. Adaption of this education program for other members of a CO-MDT, including pharmacists, may be a feasible mechanism to help further their education in CO.

Providing nurses and pharmacists with opportunities to pursue a six-to-twelve-month fellowship in CO, mirroring programs established for medical practitioners [121, 122], would help develop core clinical and leadership skills as members of the CO-MDT. Pharmacists and nurses undertaking a fellowship would need to be integrated into established CO centers under the supervision and mentorship of experienced CO clinicians; a collaborative engagement that would further strengthen the quality of CO care delivery.

The challenges, however, of implementing such education programs or fellowships in CO are duly expected. Appropriate funding, infrastructure, support from accredited CO clinicians, and strong partnerships with societal organizations are essential to feasibly integrate pharmacist and nurse training into existing CO programs. Adequate role definition for both pharmacists and nurses would also be required to ensure all members of the MDT are working within their expected scope of practice.

### The expanding cardio-oncology multidisciplinary team

The continual evolution of CO-MDTs illustrates the greater need for holistic supportive care provided by social workers, dieticians, and other allied health professionals [125]. A CO rehabilitation program for cancer survivors with high CV risk was recently established and demonstrated the positive effects of improving cardiorespiratory fitness and physical function on risk factor control and quality of life [126]. Bi-weekly exercise training sessions were conducted by a physiotherapist and supervised by a psychiatrist that incorporated both aerobic and resistance-based training [126]. Nutritional counselling from dieticians, psychologist support, and CV risk assessment have been acknowledged as key extensions for CO rehabilitation programs to further enhance cancer survivor quality of life [127]. These highlight key additions to any CO-MDT to provide more complete management of cancer survivors during and after treatment.

## Conclusion

Dedicated cardio-oncology programs are becoming widespread in the United States and Europe, mainly in academic centers, yet they are not mandated in all cancer centers. Whilst the need for comprehensive cardiac care at all stages of the cancer journey has been articulated, limited guidance has been given as to how this should be conducted. The introduction of a multidisciplinary, team-based approach offers a potential solution. CV complications of cancer treatment are heterogeneous in nature and are impacted by the presence and exacerbated by underlying risk factors and co-morbidities. Multidisciplinary and team-based strategies that promote adherence to guideline-based therapy, clinical assessment and surveillance, guideline adherence, diagnosis, patient education and empowerment may offer improvements to patient care.

Pharmacists and Nursing are well positioned to provide essential monitoring for, and management of, CTR-CVT. Increasing the utilisation of pharmacists and their repertoire of clinical skills in medication management and PMRs would be a positive contribution to a patient’s CO-MDT. It is evident that increased cooperation between cardiology and oncology teams is imperative for a true CO-MDT. Continuing education, research, and training in the space of CO would promote collaboration between healthcare professionals leading to significantly better prognoses for the patients in terms of longevity and quality of life.

## Data Availability

No datasets were generated or analysed during the current study.

## Declarations

### Précis

Integration of pharmacists and nurses into a cardio-oncology multidisciplinary team can contribute to positive patient outcomes. Further role clarification and education for pharmacists and nurses may be required to develop a true cardio-oncology multidisciplinary team.

## Abbreviations

ACEi/ARB: Angiotensin converting enzyme inhibitor/angiotensin receptor blocker
AF: Atrial fibrillation
AHA: American Heart Association
ALK: Anaplastic lymphoma kinase
ASCO: American Society of Clinical Oncology
CO: Cardio-oncology
CO-MDT: Cardio-oncology multidisciplinary team
CTR-CVT: Cancer therapy-related cardiovascular toxicity
CV: Cardiovascular
CVD: Cardiovascular disease
CYP: Cytochrome P450
DDI: Drug-drug interaction
DM: Diabetes mellitus
ESC: European Society of Cardiology
ESMO: European Society of Medical Oncology
HF: Heart failure
HTM: Hypertension
iCOP-pharm: Iraqi Cardio-Oncology Program-Pharmacist program
IC-OS: International Society of Cardio-Oncology
LV: left ventricle
NP: Nurse practitioner
P-gp: P-glycoprotein
PMR: Pharmacist-led medication review
TITAN: Team IntervenTion in cArdio-oNcology trial

## References

[1] Naghavi M, Ong KL, Aali A, Ababneh HS, Abate YH, Abbafati C, et al. Global burden of 288 causes of death and life expectancy decomposition in 204 countries and territories and 811 subnational locations, 1990–2021: a systematic analysis for the global burden of disease study 2021. Lancet. 2024;403(10440):2100–32. 38582094 10.1016/S0140-6736(24)00367-2 PMC11126520

[2] International Agency for Research on Cancer. Global Cancer Observatory Lyon, France: World Health Organization; 2024 [cited 2024 Oct 03]. Available from: https://gco.iarc.fr/en

[3] Cancer Council Australia. Australians living with and beyond cancer in 2040. Victoria: Cancer Council Australia; 2018.

[4] Miller KD, Nogueira L, Devasia T, Mariotto AB, Yabroff KR, Jemal A, et al. Cancer treatment and survivorship statistics, 2022. Cancer J Clin. 2022;72(5):409–36. 10.3322/caac.21731 35736631

[5] Siegel RL, Giaquinto AN, Jemal A. Cancer statistics, 2024. Cancer J Clin. 2024;74(1):12–49. 10.3322/caac.21820 38230766

[6] Mani K, Deng D, Lin C, Wang M, Hsu ML, Zaorsky NG. Causes of death among people living with metastatic cancer. Nat Commun. 2024;15(1):1519. 38374318 10.1038/s41467-024-45307-x PMC10876661

[7] Ngo DT, Williams T, Horder S, Kritharides L, Vardy J, Mandaliya H, et al. Factors associated with adverse cardiovascular events in cancer patients treated with bevacizumab. J Clin Med. 2020;9(8):2664. 32824667 10.3390/jcm9082664 PMC7465018

[8] Curigliano G, Cardinale D, Dent S, Criscitiello C, Aseyev O, Lenihan D, et al. Cardiotoxicity of anticancer treatments: epidemiology, detection, and management. Cancer J Clin. 2016;66(4):309–25. 10.3322/caac.21341 26919165

[9] Lyon AR, Dent S, Stanway S, Earl H, Brezden-Masley C, Cohen‐Solal A, et al. Baseline cardiovascular risk assessment in cancer patients scheduled to receive cardiotoxic cancer therapies: a position statement and new risk assessment tools from the C ardio‐O ncology S Tudy G Roup of the H eart F ailure A ssociation of the E uropean S ociety of C ardiology in collaboration with the I nternational C ardio‐O ncology S ociety. Eur J Heart Fail. 2020;22(11):1945–60. 32463967 10.1002/ejhf.1920 PMC8019326

[10] Lyon AR, López-Fernández T, Couch LS, Asteggiano R, Aznar MC, Bergler-Klein J, et al. 2022 ESC guidelines on cardio-oncology developed in collaboration with the European hematology association (EHA), the European society for therapeutic radiology and oncology (ESTRO) and the international Cardio-Oncology society (IC-OS) developed by the task force on cardio-oncology of the European society of cardiology (ESC). Eur Heart J. 2022;43(41):4229–361. 36017568 10.1093/eurheartj/ehac244

[11] Herrmann J, Lenihan D, Armenian S, Barac A, Blaes A, Cardinale D, et al. Defining cardiovascular toxicities of cancer therapies: an international Cardio-Oncology society (IC-OS) consensus statement. Eur Heart J. 2022;43(4):280–99. 34904661 10.1093/eurheartj/ehab674 PMC8803367

[12] Curigliano G, Lenihan D, Fradley M, Ganatra S, Barac A, Blaes A, et al. Management of cardiac disease in cancer patients throughout oncological treatment: ESMO consensus recommendations. Ann Oncol. 2020;31(2):171–90. 31959335 10.1016/j.annonc.2019.10.023 PMC8019325

[13] Armenian SH, Lacchetti C, Barac A, Carver J, Constine LS, Denduluri N, et al. Prevention and monitoring of cardiac dysfunction in survivors of adult cancers: American society of clinical oncology clinical practice guideline. J Clin Oncol. 2017;35(8):893–911. 27918725 10.1200/JCO.2016.70.5400

[14] Kourek C, Touloupaki M, Rempakos A, Loritis K, Tsougkos E, Paraskevaidis I, et al. Cardioprotective strategies from cardiotoxicity in Cancer patients: A comprehensive review. J Cardiovasc Dev Disease. 2022;9(8):259. 36005423 10.3390/jcdd9080259 PMC9409997

[15] Herrmann J. Adverse cardiac effects of cancer therapies: cardiotoxicity and arrhythmia. Nat Reviews Cardiol. 2020;17(8):474–502. 10.1038/s41569-020-0348-1 PMC8782611 32231332

[16] Sarfati D, Koczwara B, Jackson C. The impact of comorbidity on cancer and its treatment. Cancer J Clin. 2016;66(4):337–50. 10.3322/caac.21342 26891458

[17] Leach CR, Weaver KE, Aziz NM, Alfano CM, Bellizzi KM, Kent EE, et al. The complex health profile of long-term cancer survivors: prevalence and predictors of comorbid conditions. J Cancer Surviv. 2015;9:239–51. 25319681 10.1007/s11764-014-0403-1

[18] Scotté F, Taylor A, Davies A. Supportive care: the keystone of modern oncology practice. Cancers. 2023;15(15):3860. 37568675 10.3390/cancers15153860 PMC10417474

[19] Parent S, Pituskin E, Paterson DI. The Cardio-oncology program: A multidisciplinary approach to the care of Cancer patients with cardiovascular disease. Can J Cardiol. 2016;32(7):847–51. 27343743 10.1016/j.cjca.2016.04.014

[20] Lancellotti P, Suter TM, López-Fernández T, Galderisi M, Lyon AR, Van der Meer P, et al. Cardio-oncology services: rationale, organization, and implementation: a report from the ESC Cardio-Oncology Council. Eur Heart J. 2019;40(22):1756–63. 30085070 10.1093/eurheartj/ehy453

[21] Coopers P. The economic costs of atrial fibrillation in Australia. National Stroke Foundation; 2010.

[22] Hendriks JM, De Wit R, Crijns HJ, Vrijhoef HJ, Prins MH, Pisters R, et al. Nurse-led care vs. usual care for patients with atrial fibrillation: results of a randomized trial of integrated chronic care vs. routine clinical care in ambulatory patients with atrial fibrillation. Eur Heart J. 2012;33(21):2692–9. 22453654 10.1093/eurheartj/ehs071

[23] Hendriks J, Tomini F, Van Asselt T, Crijns H, Vrijhoef H. Cost-effectiveness of a specialized atrial fibrillation clinic vs. usual care in patients with atrial fibrillation. Europace. 2013;15(8):1128–35. 23515338 10.1093/europace/eut055

[24] Dunn SP, Birtcher KK, Beavers CJ, Baker WL, Brouse SD, Page RL, et al. The role of the clinical pharmacist in the care of patients with cardiovascular disease. J Am Coll Cardiol. 2015;66(19):2129–39. 26541925 10.1016/j.jacc.2015.09.025

[25] Lau DH, Nattel S, Kalman JM, Sanders P. Modifiable risk factors and atrial fibrillation. Circulation. 2017;136(6):583–96. 28784826 10.1161/CIRCULATIONAHA.116.023163

[26] Hendriks JM, Jaarsma T. The multidisciplinary team approach in cardiovascular care. Eur J Cardiovasc Nurs. 2020;20(2):91–2. 10.1093/eurjcn/zvaa005 33620471

[27] Ski CF, Cartledge S, Foldager D, Thompson DR, Fredericks S, Ekman I, et al. Integrated care in cardiovascular disease: a statement of the association of cardiovascular nursing and allied professions of the European society of cardiology. Eur J Cardiovasc Nurs. 2023;22(5):e39–46. 36617217 10.1093/eurjcn/zvad009

[28] Bray F, Laversanne M, Sung H, Ferlay J, Siegel RL, Soerjomataram I, et al. Global cancer statistics 2022: GLOBOCAN estimates of incidence and mortality worldwide for 36 cancers in 185 countries. Cancer J Clin. 2024;74(3):229–63. 10.3322/caac.21834 38572751

[29] Health, AIo. Welfare. Cancer data in Australia. Canberra: AIHW; 2024.

[30] Siegel RL, Giaquinto AN, Jemal A, Cancer statistics. 2024. CA: a cancer journal for clinicians. 2024;74(1). 10.3322/caac.21820 38230766

[31] Spinewine A, Fialová D, Byrne S. The role of the pharmacist in optimizing pharmacotherapy in older people. Drugs Aging. 2012;29:495–510. 22642783 10.2165/11631720-000000000-00000

[32] Martin P, Tamblyn R, Benedetti A, Ahmed S, Tannenbaum C. Effect of a pharmacist-led educational intervention on inappropriate medication prescriptions in older adults: the D-PRESCRIBE randomized clinical trial. JAMA. 2018;320(18):1889–98. 30422193 10.1001/jama.2018.16131 PMC6248132

[33] Bukhsh A, Khan TM, Lee SW, Lee L-H, Chan K-G, Goh B-H. Efficacy of pharmacist based diabetes educational interventions on clinical outcomes of adults with type 2 diabetes mellitus: a network meta-analysis. Front Pharmacol. 2018;9:339. 29692730 10.3389/fphar.2018.00339 PMC5902757

[34] Jaam M, Naseralallah LM, Hussain TA, Pawluk SA. Pharmacist-led educational interventions provided to healthcare providers to reduce medication errors: A systematic review and meta-analysis. PLoS ONE. 2021;16(6):e0253588. 34161388 10.1371/journal.pone.0253588 PMC8221459

[35] Avery M, Williams F. The importance of pharmacist providing patient education in oncology. J Pharm Pract. 2015;28(1):26–30. 25540194 10.1177/0897190014562382

[36] Lee KMK, Koeper I, Johnson ME, Page A, Rowett D, Johnson J. Multidisciplinary perspectives on roles of hospital pharmacists in tertiary settings: a qualitative study. Int J Qual Health Care. 2023;36(1). 10.1093/intqhc/mzad110 PMC10795054 38155609

[37] Snipelisky D, Park JY, Lerman A, Mulvagh S, Lin G, Pereira N, et al. How to develop a cardio-oncology clinic. Heart Fail Clin. 2017;13(2):347–59. 28279420 10.1016/j.hfc.2016.12.011

[38] Nhola LF, Villarraga HR. Rationale for cardio-oncology units. Revista Española De Cardiología (English Edition). 2017;70(7):583–9. 10.1016/j.rec.2017.02.006 28330611

[39] Agunbiade TA, Ottaviano Y, Goswami D, Ruiz G, Barac A. Targeting barriers of systems of care in a growing Multi-disciplinary field. Curr Oncol Rep. 2019;21(4):1–8. 10.1007/s11912-019-0785-3 30859328

[40] Pituskin E, Haykowsky M, McNeely M, Mackey J, Chua N, Paterson I. Rationale and design of the multidisciplinary team intervention in cArdio-oNcology study (TITAN). BMC Cancer. 2016;16(1):733. 27629548 10.1186/s12885-016-2761-8 PMC5024526

[41] ClinicalTrials.gov. Identifier NCT01621659, Multidisciplinary Team IntervenTion in CArdio-ONcology (TITAN Study) (TITAN) Bethesda, MD: National Library of Medicine, US; 2012 [updated 19th Jul 2024; cited 2024 2nd Dec]. Available from: https://clinicaltrials.gov/study/NCT01621659

[42] Brown S-A, Patel S, Rayan D, Zaharova S, Lin M, Nafee T, et al. A virtual-hybrid approach to launching a cardio-oncology clinic during a pandemic. Cardiooncology. 2021;7(1):2. 33441188 10.1186/s40959-020-00088-2 PMC7803880

[43] Warden BA, Shapiro MD, Fazio S. The role of the clinical pharmacist in a preventive cardiology practice. Ann Pharmacother. 2019;53(12):1214–9. 31342786 10.1177/1060028019864669

[44] Ma CSJ. Role of pharmacists in optimizing the use of anticancer drugs in the clinical setting. Integr Pharm Res Pract. 2014;3(null):11–24.

[45] Holle LM, Segal EM, Jeffers KD. The expanding role of the oncology pharmacist. Pharmacy. 2020;8(3):130. 32722357 10.3390/pharmacy8030130 PMC7557441

[46] Merali A, Anwar M, Boyd JM, McFarlane T, Daniluk M. Exploration of current pharmacy practice in cardio-oncology: experiences & perspectives. J Oncol Pharm Pract. 2023;29(8):1844–52. 36537037 10.1177/10781552221145667

[47] Patel S, Dushenkov A, Jungsuwadee P, Krishnaswami A, Barac A. Team-based approach to management of hypertension associated with angiogenesis inhibitors. J Cardiovasc Transl Res. 2020;13(3):463–77. 32430701 10.1007/s12265-020-10024-5

[48] Wimmer BC, Cross AJ, Jokanovic N, Wiese MD, George J, Johnell K, et al. Clinical outcomes associated with medication regimen complexity in older people: a systematic review. J Am Geriatr Soc. 2017;65(4):747–53. 27991653 10.1111/jgs.14682

[49] Pantuzza LL, Ceccato MGB, Silveira MR, Junqueira LMR, Reis AMM. Association between medication regimen complexity and pharmacotherapy adherence: a systematic review. Eur J Clin Pharmacol. 2017;73(11):1475–89. 28779460 10.1007/s00228-017-2315-2

[50] Lisy K, Kent J, Piper A, Jefford M. Facilitators and barriers to shared primary and specialist cancer care: a systematic review. Support Care Cancer. 2021;29:85–96. 32803729 10.1007/s00520-020-05624-5

[51] Clark RA, Marin TS, McCarthy AL, Bradley J, Grover S, Peters R, et al. Cardiotoxicity after cancer treatment: a process map of the patient treatment journey. Cardio-Oncology. 2019;5:1–11. 32154020 10.1186/s40959-019-0046-5 PMC7048085

[52] Viswanathan M, Kahwati LC, Golin CE, Blalock SJ, Coker-Schwimmer E, Posey R, et al. Medication therapy management interventions in outpatient settings: a systematic review and meta-analysis. JAMA Intern Med. 2015;175(1):76–87. 25401788 10.1001/jamainternmed.2014.5841

[53] Jokanovic N, Tan EC, Sudhakaran S, Kirkpatrick CM, Dooley MJ, Ryan-Atwood TE, et al. Pharmacist-led medication review in community settings: an overview of systematic reviews. Res Social Administrative Pharm. 2017;13(4):661–85. 10.1016/j.sapharm.2016.08.005 27665364

[54] Jokanovic N, Tan ECK, Sudhakaran S, Kirkpatrick CM, Dooley MJ, Ryan-Atwood TE, et al. Pharmacist-led medication review in community settings: an overview of systematic reviews. Res Social Administrative Pharm. 2017;13(4):661–85. 10.1016/j.sapharm.2016.08.005 27665364

[55] Stewart D, Whittlesea C, Dhital R, Newbould L, McCambridge J. Community pharmacist led medication reviews in the UK: a scoping review of the medicines use review and the new medicine service literatures. Res Social Administrative Pharm. 2020;16(2):111–22. 10.1016/j.sapharm.2019.04.010 31085141

[56] Deng Z-J, Gui L, Chen J, Peng S-S, Ding Y-F, Wei A-H. Clinical, economic and humanistic outcomes of medication therapy management services: A systematic review and meta-analysis. Front Pharmacol. 2023;14:1143444. 37089963 10.3389/fphar.2023.1143444 PMC10113465

[57] Pharmaceutical Society of New Zealand. Medicines Use Review Wellington, New Zealand: Pharmaceutical Society of New Zealand; 2024 [cited 2024 Oct 10]. Available from: https://www.psnz.org.nz/healthservices/mur#:~:text=Pharmacy%20Healthcare%20Services%26text=If%20you%20take%20multiple%20long,getting%20maximum%20benefit%20from%20them

[58] Bennetts J, White J, Croft H, Cooper J, McIvor D, Eadie N, et al. Pharmacist-led medication management services: a qualitative exploration of transition-of-care cardiovascular disease patient experiences. BMJ Open. 2024;14(5):e082228. 10.1136/bmjopen-2023-082228 PMC11116877 38777587

[59] Ismail M, Khan S, Khan F, Noor S, Sajid H, Yar S, et al. Prevalence and significance of potential drug-drug interactions among cancer patients receiving chemotherapy. BMC Cancer. 2020;20:1–9. 10.1186/s12885-020-06855-9 PMC7168989 32307008

[60] Beavers CJ, Rodgers JE, Bagnola AJ, Beckie TM, Campia U, Di Palo KE, et al. Cardio-Oncology drug interactions: A scientific statement from the American heart association. Circulation. 2022;145(15):e811–38. 35249373 10.1161/CIR.0000000000001056

[61] Fatunde OA, Brown S-A. The role of CYP450 drug metabolism in precision Cardio-Oncology. Int J Mol Sci. 2020;21(2):604. 31963461 10.3390/ijms21020604 PMC7014347

[62] Riechelmann RP, Krzyzanowska MK. Drug interactions and oncological outcomes: a hidden adversary. Ecancermedicalscience. 2019;13:ed88. 31123502 10.3332/ecancer.2019.ed88 PMC6467454

[63] Mosarla RC, Vaduganathan M, Qamar A, Moslehi J, Piazza G, Giugliano RP. Anticoagulation strategies in patients with cancer: JACC review topic of the week. J Am Coll Cardiol. 2019;73(11):1336–49. 30898209 10.1016/j.jacc.2019.01.017 PMC7957366

[64] Dziadziuszko R, Peters S, Ruf T, Cardona A, Guerini E, Kurtsikidze N, et al. Clinical experience and management of adverse events in patients with advanced ALK-positive non-small-cell lung cancer receiving alectinib. ESMO Open. 2022;7(6):100612. 36375271 10.1016/j.esmoop.2022.100612 PMC9663323

[65] Porta-Sánchez A, Gilbert C, Spears D, Amir E, Chan J, Nanthakumar K, et al. Incidence, diagnosis, and management of QT prolongation induced by Cancer therapies: A systematic review. J Am Heart Association. 2017;6(12):e007724. 10.1161/JAHA.117.007724 PMC5779062 29217664

[66] Kuan PX, Chan WK, Ying DKF, Rahman MAA, Peariasamy KM, Lai NM, et al. Efficacy of telemedicine for the management of cardiovascular disease: a systematic review and meta-analysis. Lancet Digit Health. 2022;4(9):e676–91. 36028290 10.1016/S2589-7500(22)00124-8 PMC9398212

[67] Subedi N, Rawstorn JC, Gao L, Koorts H, Maddison R. Implementation of telerehabilitation interventions for the self-management of cardiovascular disease: systematic review. JMIR mHealth uHealth. 2020;8(11):e17957. 33245286 10.2196/17957 PMC7732711

[68] McGrowder DA, Miller FG, Vaz K, Anderson Cross M, Anderson-Jackson L, Bryan S, et al. editors. The utilization and benefits of telehealth services by health care professionals managing breast cancer patients during the COVID-19 pandemic. Healthcare: MDPI; 2021. 10.3390/healthcare9101401 PMC8535379 34683081

[69] Zon RT, Kennedy EB, Adelson K, Blau S, Dickson N, Gill D, et al. Telehealth in oncology: ASCO standards and practice recommendations. JCO Oncol Pract. 2021;17(9):546–64. 34319760 10.1200/OP.21.00438

[70] Batalik L, Filakova K, Radkovcova I, Dosbaba F, Winnige P, Vlazna D, et al. Cardio-oncology rehabilitation and telehealth: rationale for future integration in supportive care of cancer survivors. Front Cardiovasc Med. 2022;9:858334. 35497988 10.3389/fcvm.2022.858334 PMC9051023

[71] Sankaranarayanan J, Murante LJ, Moffett LM. A retrospective evaluation of remote pharmacist interventions in a telepharmacy service model using a conceptual framework. Telemedicine e-Health. 2014;20(10):893–901. 24611489 10.1089/tmj.2013.0362 PMC4188381

[72] Crilly P, Kayyali R. A systematic review of randomized controlled trials of telehealth and digital technology use by community pharmacists to improve public health. Pharmacy. 2020;8(3):137. 32759850 10.3390/pharmacy8030137 PMC7559081

[73] Zamorano J. An ESC position paper on cardio-oncology. Eur Heart J. 2016;37(36):2739–40. 27694539 10.1093/eurheartj/ehw359

[74] New JP, Mason JM, Freemantle N, Teasdale S, Wong LM, Bruce NJ, et al. Specialist nurse-led intervention to treat and control hypertension and hyperlipidemia in diabetes (SPLINT): a randomized controlled trial. Diabetes Care. 2003;26(8):2250–5. 12882844 10.2337/diacare.26.8.2250

[75] McDonald VM, Vertigan AE, Gibson PG. How to set up a severe asthma service. Respirology. 2011;16(6):900–11. 21692918 10.1111/j.1440-1843.2011.02012.x

[76] Doherty M, Jenkins W, Richardson H, Sarmanova A, Abhishek A, Ashton D, et al. Efficacy and cost-effectiveness of nurse-led care involving education and engagement of patients and a treat-to-target urate-lowering strategy versus usual care for gout: a randomised controlled trial. Lancet. 2018;392(10156):1403–12. 30343856 10.1016/S0140-6736(18)32158-5 PMC6196879

[77] Hendriks JML, de Wit R, Crijns HJGM, Vrijhoef HJM, Prins MH, Pisters R, et al. Nurse-led care vs. usual care for patients with atrial fibrillation: results of a randomized trial of integrated chronic care vs. routine clinical care in ambulatory patients with atrial fibrillation. Eur Heart J. 2012;33(21):2692–9. 22453654 10.1093/eurheartj/ehs071

[78] Dalal HM, Doherty P, Taylor RS. Cardiac rehabilitation. BMJ: Br Med J. 2015;351:h5000. 26419744 10.1136/bmj.h5000 PMC4586722

[79] Stewart S, Wiley JF, Ball J, Chan YK, Ahamed Y, Thompson DR, et al. Impact of Nurse-Led, multidisciplinary Home-Based intervention on Event-Free survival across the spectrum of chronic heart disease: composite analysis of health outcomes in 1226 patients from 3 randomized trials. Circulation. 2016;133(19):1867–77. 27083509 10.1161/CIRCULATIONAHA.116.020730 PMC4857795

[80] Al-Mallah MH, Farah I, Al-Madani W, Bdeir B, Al Habib S, Bigelow ML, et al. The impact of Nurse-Led clinics on the mortality and morbidity of patients with cardiovascular diseases: A systematic review and Meta-analysis. J Cardiovasc Nurs. 2016;31(1):89–95. 25658181 10.1097/JCN.0000000000000224

[81] Rush KL, Burton L, Schaab K, Lukey A. The impact of nurse-led atrial fibrillation clinics on patient and healthcare outcomes: a systematic mixed studies review. Eur J Cardiovasc Nurs. 2019;18(7):526–33. 31046431 10.1177/1474515119845198

[82] Brieger D, Amerena J, Attia J, Bajorek B, Chan KH, Connell C, National Heart Foundation of Australia and the Cardiac Society of Australia and New Zealand. Australian clinical guidelines for the diagnosis and management of atrial fibrillation 2018. Heart Lung Circ. 2018;27(10):1209–66. 30077228 10.1016/j.hlc.2018.06.1043

[83] Hindricks G, Potpara T, Dagres N, Arbelo E, Bax JJ, Blomström-Lundqvist C, et al. 2020 ESC guidelines for the diagnosis and management of atrial fibrillation developed in collaboration with the European association for Cardio-Thoracic surgery (EACTS): the task force for the diagnosis and management of atrial fibrillation of the European society of cardiology (ESC) developed with the special contribution of the European heart rhythm association (EHRA) of the ESC. Eur Heart J. 2020;42(5):373–498. 10.1093/eurheartj/ehaa612 32860505

[84] McDonagh TA, Metra M, Adamo M, Gardner RS, Baumbach A, Böhm M, et al. 2021 ESC guidelines for the diagnosis and treatment of acute and chronic heart failure: developed by the task force for the diagnosis and treatment of acute and chronic heart failure of the European society of cardiology (ESC) with the special contribution of the heart failure association (HFA) of the ESC. Eur Heart J. 2021;42(36):3599–726.34447992

[85] David D, Britting L, Dalton J. Cardiac acute care nurse practitioner and 30-day readmission. J Cardiovasc Nurs. 2015;30(3):248–55. 24651684 10.1097/JCN.0000000000000147

[86] Driscoll A, Meagher S, Kennedy R, Hare DL, Johnson DF, Asker K, et al. Impact of a heart failure nurse practitioner service on rehospitalizations, emergency presentations, and survival in patients hospitalized with acute heart failure. Eur J Cardiovasc Nurs. 2022;22(7):701–8. 10.1093/eurjcn/zvac108 36413653

[87] O’Toole J, Ingram S, Kelly N, Quirke MB, Roberts A, O’Brien F. Patient Satisfaction With Innovative Nurse Practitioner Cardiology Services. The Journal for Nurse Practitioners. 2019;15(4):311-5.e1.

[88] Smigorowsky MJ, Sebastianski M, Sean McMurtry M, Tsuyuki RT, Norris CM. Outcomes of nurse practitioner-led care in patients with cardiovascular disease: A systematic review and meta‐analysis. J Adv Nurs. 2020;76(1):81–95. 31588598 10.1111/jan.14229 PMC6973236

[89] Oncology Nursing Society. Oncology nurse practitioner competencies. Pittsburgh, PA: Oncology Nursing Society; 2007.

[90] Coombs LA, Noonan K, Diane Barber F, Mackey H, Peterson ME, Turner T, et al. Oncology nurse practitioner competencies: defining best practices in the oncology setting. Clin J Oncol Nurs. 2020;24(3):296–304. 32441678 10.1188/20.CJON.296-304 PMC8152155

[91] van Dusseldorp L, Groot M, Adriaansen M, van Vught A, Vissers K, Peters J. What does the nurse practitioner mean to you? A patient-oriented qualitative study in oncological/palliative care. J Clin Nurs. 2019;28(3–4):589–602. 30129072 10.1111/jocn.14653 PMC7380134

[92] McCavery A. A nurse Practitioner-Led model to implement and deliver unscheduled emergency Cancer care. J Nurse Practitioners. 2020;16(4):271–5.

[93] Oatley M, Fry M. A nurse practitioner–led model of care improves access, early assessment and integration of oncology services: an evaluation study. Support Care Cancer. 2020;28(10):5023–9. 32040635 10.1007/s00520-019-05292-0

[94] Cox KOAMRNONP, Karikios DBMF, Roydhouse JKBAMPH, White KRNCCM. Nurse-led supportive care management: a 6-month review of the role of a nurse practitioner in a chemotherapy unit. Aust Health Rev. 2013;37(5):632–5. 24176186 10.1071/AH13069

[95] Thom B, Boekhout AH, Corcoran S, Adsuar R, Oeffinger KC, McCabe MS. Advanced practice providers and survivorship care: they can deliver. J Oncol Pract. 2019;15(3):e230–7. 30615587 10.1200/JOP.18.00359 PMC7850669

[96] Barros-Gomes S, Herrmann J, Mulvagh SL, Lerman A, Lin G, Villarraga HR. Rationale for setting up a cardio-oncology unit: our experience at Mayo clinic. Cardio-Oncology. 2016;2:1–9. 33530144 10.1186/s40959-016-0014-2 PMC7837147

[97] Fadol A. Cardio-oncology nursing. Practical Cardio-Oncology: CRC; 2019. pp. 189–204.

[98] Berra K, Miller NH, Jennings C. Nurse-based models for cardiovascular disease prevention: from research to clinical practice. Eur J Cardiovasc Nursing: J Working Group Cardiovasc Nurs Eur Soc Cardiol. 2011;10(Suppl 2):S42–50. 10.1016/S1474-5151(11)00115-0 21762851

[99] Dorst DCHv, Dobbin SJH, Neves KB, Herrmann J, Herrmann SM, Versmissen J, et al. Hypertension and prohypertensive antineoplastic therapies in Cancer patients. Circul Res. 2021;128(7):1040–61. 10.1161/CIRCRESAHA.121.318051 PMC8011349 33793337

[100] Potter PA, Perry AG, Stockert PA, Hall A. Fundamentals of nursing-e-book. Elsevier health sciences; 2021.

[101] Carter BL, Rogers M, Daly J, Zheng S, James PA. The potency of team-based care interventions for hypertension: a meta-analysis. Arch Intern Med. 2009;169(19):1748–55. 19858431 10.1001/archinternmed.2009.316 PMC2882164

[102] Fazel MT, Bagalagel A, Lee JK, Martin JR, Slack MK. Impact of diabetes care by pharmacists as part of health care team in ambulatory settings: A systematic review and Meta-analysis. Annals Pharmacotherapy. 2017;51(10):890–907. 10.1177/1060028017711454 28573873

[103] Fadol A, Estrella J, Shelton V, Zaghian M, Vanbenschop D, Counts V, et al. A quality improvement approach to reducing hospital readmissions in patients with cancer and heart failure. Cardiooncology. 2019;5:5. 32154012 10.1186/s40959-019-0041-x PMC7048036

[104] Zirkelbach R, Skurka K, Rao VU. A nurse navigator led Community-Based Cardio-Oncology clinic. Oncol Issues. 2021;36(3):50–8.

[105] Yaseen IF, Farhan HA. Cardiovascular drug interventions in the cardio-oncology clinic by a cardiology pharmacist: iCOP-pharm study. Front Cardiovasc Med. 2022;9:972455. 36247485 10.3389/fcvm.2022.972455 PMC9556995

[106] Einsfeld L, do Canto Olegário I, Fagundes ML. Intersecting care through specialized pharmacists: A case report of residency rotation focused on the new horizon of cardio-oncology. Currents Pharm Teach Learn. 2023;15(5):508–13. 10.1016/j.cptl.2023.04.018 37183144

[107] Pareek N, Cevallos J, Moliner P, Shah M, Tan LL, Chambers V, et al. Activity and outcomes of a cardio-oncology service in the united Kingdom—a five‐year experience. Eur J Heart Fail. 2018;20(12):1721–31. 30191649 10.1002/ejhf.1292

[108] Zhang C, Yang Z, Du R, Feng Y, Zhang X, Zhang J. Cardio-oncologic knowledge of nurses in the oncology service: a multi-center survey in China. J Multidisciplinary Healthc. 2023:4027–38. 10.2147/JMDH.S436376 PMC10727691 38111828

[109] Liu Y. A novel cardio-oncology service line model in optimizing care access, quality and equity for large, multi-hospital health systems. Cardio-Oncology. 2023;9(1):16. 36973756 10.1186/s40959-023-00167-0 PMC10041762

[110] Brieger DB, Redfern J. Contemporary themes in acute coronary syndrome management: from acute illness to secondary prevention. Med J Aust. 2013;199(3):174–8. 23909538 10.5694/mja12.11224

[111] Fox P, Boyce A. Cancer health inequality persists in regional and remote Australia. Communities. 2014;83:02literaturereviewmodelscancerservicesruralandremote. 10.5694/mja14.01217 25332023

[112] Walsh WF, Kangaharan N. Cardiac care for Indigenous australians: practical considerations from a clinical perspective. Med J Aust. 2017;207(1):40–5. 28659116 10.5694/mja17.00250

[113] Arnold RH, Tideman PA, Devlin GP, Carroll GE, Elder A, Lowe H, et al. Rural and remote cardiology during the COVID-19 pandemic: cardiac society of Australia and new Zealand (CSANZ) consensus statement. Heart Lung Circulation. 2020;29(7):e88–93. 32487432 10.1016/j.hlc.2020.05.001 PMC7203036

[114] Taylor D, van der Merwe V, van der Merwe W. Nurse Titration clinics to achieve rapid control of blood pressure. NZ Med J. 2012;125(1355):31–40. 22722213

[115] Cui X, Zhou X, Ma LL, Sun TW, Bishop L, Gardiner FW, et al. A nurse-led structured education program improves self-management skills and reduces hospital readmissions in patients with chronic heart failure: a randomized and controlled trial in China. Rural Remote Health. 2019;19(2):5270. 31113205 10.22605/RRH5270

[116] McLachlan A, Aldridge C, Lee M, Harper C, Kerr A. The development and first six years of a nurse-led chest pain clinic. N Z Med J. 2019;132(1489):39–47. 30703778

[117] Ramanath KV, Balaji DBSS, Nagakishore CH, Mahesh Kumar S, Bhanuprakash M. A study on impact of clinical pharmacist interventions on medication adherence and quality of life in rural hypertensive patients. J Young Pharmacists. 2012;4(2):95–100. 10.4103/0975-1483.96623 PMC3385224 22754261

[118] Forough AS, Esfahani PR. Impact of pharmacist intervention on appropriate insulin pen use in older patients with type 2 diabetes mellitus in a rural area in Iran. J Res Pharm Pract. 2017;6(2):114. 28616435 10.4103/jrpp.JRPP_16_151 PMC5463546

[119] Falamić S, Lucijanić M, Ortner-Hadžiabdić M, Marušić S, Bačić-Vrca V. Pharmacists’ interventions improve health-related quality of life of rural older person on warfarin: a randomized controlled trial. Sci Rep. 2021;11(1):1–7. 34754004 10.1038/s41598-021-01394-0 PMC8578616

[120] Lenihan DJ, Hartlage G, DeCara J, Blaes A, Finet JE, Lyon AR, et al. Cardio-Oncology training: A proposal from the international cardioncology society and Canadian cardiac oncology network for a new multidisciplinary specialty. J Card Fail. 2016;22(6):465–71. 27038642 10.1016/j.cardfail.2016.03.012

[121] Alvarez-Cardona JA, Ray J, Carver J, Zaha V, Cheng R, Yang E, et al. Cardio-Oncology education and training. J Am Coll Cardiol. 2020;76(19):2267–81. 33153587 10.1016/j.jacc.2020.08.079 PMC8174559

[122] Tuzovic M, Brown S-A, Yang EH, West BH, Bassi NS, Park S, et al. Implementation of Cardio-Oncology training for cardiology fellows. JACC: CardioOncology. 2020;2(5):795–9. 34396296 10.1016/j.jaccao.2020.11.003 PMC8352041

[123] Brown SA, Yang EH, Branch M, Beavers C, Blaes A, Fradley MG, et al. Training and career development in Cardio-Oncology translational and implementation science. Heart Fail Clin. 2022;18(3):503–14. 35718422 10.1016/j.hfc.2022.02.014 PMC9236555

[124] International Cardio-Oncology Society, Tampa FL. IC-OS; 2024 [cited 2024 Nov 05]. Available from: https://ic-os.org/new/

[125] Fadol AP, Palaskas NL, Ewer MS, Deswal A. An overview of a different type of cardio-oncology gathering: summary of the COMP (cardio-oncology multidisciplinary practice) meeting held in Houston texas, January 2020. Cardio-Oncology. 2020;6(1):20. 32944289 10.1186/s40959-020-00075-7 PMC7490902

[126] Viamonte SG, Joaquim AV, Alves AJ, Vilela E, Capela A, Ferreira C, et al. Cardio-Oncology rehabilitation for Cancer survivors with high cardiovascular risk: A randomized clinical trial. JAMA Cardiol. 2023;8(12):1119–28. 37819656 10.1001/jamacardio.2023.3558 PMC10568446

[127] Bisceglia I, Venturini E, Canale ML, Ambrosetti M, Riccio C, Giallauria F, et al. Cardio-oncology rehabilitation: are we ready? Eur Heart J Supplements. 2024;26(Supplement2):ii252–63. 10.1093/eurheartjsupp/suae030 PMC11110456 38784673

